# MiR-373-3p促进肺腺癌细胞的侵袭转移

**DOI:** 10.3779/j.issn.1009-3419.2015.07.07

**Published:** 2015-07-20

**Authors:** 爱兵 吴, 金媚 李, 昆鹏 吴, 艳丽 莫, 怡平 罗, 海茵 叶, 湘 沈, 姝君 李, 亚海 梁, 美莲 刘, 志雄 杨

**Affiliations:** 524000 湛江，广东医学院附属医院肿瘤科 Department of Oncology, Affiliated Hospital of Guangdong Medical College, Zhanjiang 524000, China

**Keywords:** 肺肿瘤, MiR-373, MMP-9, MMP-14, 侵袭, 转移, Lung neoplasms, MiR-373, MMP-9, MMP-14, Invasion, Metastasis

## Abstract

**背景与目的:**

肺癌位居全球癌症相关死亡率的首位，其中肿瘤转移是导致肺癌患者死亡的主要原因，研究表明miR-373与多种肿瘤细胞的侵袭转移有密切关系。本研究旨在探讨miR-373-3p在非小细胞肺癌(non-small cell lung cancer, NSCLC)中的表达情况及其对肺腺癌细胞侵袭转移能力的影响。

**方法:**

利用qRT-PCR法检测miR-373-3p在NSCLC组织和肺腺癌细胞株中的表达。瞬时转染hsa-miR-373-3p的mimics和inhibitor至肺腺癌H1299和A549细胞株中，利用Transwell小室检测转染后肺腺癌细胞侵袭转移能力的改变，Western blot检测转染后肺腺癌细胞中基质金属蛋白酶-9(matrix metalloproteinase-9, MMP-9)及MMP-14蛋白水平的改变。

**结果:**

miR-373-3p在51例NSCLC组织和5种肺腺癌细胞株中均明显高表达。在miR-373-3p低表达的H1299细胞中过表达miR-373-3p，细胞的侵袭转移能力明显提高，同时MMP-9及MMP-14的表达上调；在miR-373-3p高表达的A549细胞中抑制miR-373-3p表达，细胞的侵袭转移能力下降，并且下调MMP-9和MMP-14的表达。

**结论:**

miR-373-3p可能通过正向调节MMP-9、MMP-14的表达而促进肺腺癌细胞的侵袭转移能力。

## Abstract (en)

**Background and objective:**

Lung cancer is the leading cause of cancer-related deaths worldwide, and metastasis is the major cause of death in lung cancer patients. MiR-373 is closely associated with invasion and metastasis in other tumor cells. This study explored the expression of miR-373-3p in non-small cell lung cancer (NSCLC) and its effect on the invasive and metastatic capabilities of lung adenocarcinoma cells, as well as their mechanisms of action.

**Methods:**

The expression of miR-373-3p in NSCLC tissues and lung adenocarcinoma cell lines was detected by quantitative reverse transcription polymerase chain reaction. The roles of miR-373-3p in regulating lung adenocarcinoma cell invasion and metastatic properties were analyzed with miR-373-3p mimic/inhibitor-transfected cells via Transwell chamber assay. Matrix metalloproteinase MMP-9 and MMP-14 protein levels were detected by Western blot in lung cancer cells after transfection.

**Results:**

MiR-373-3p was upregulated in 51 NSCLC tissues and 5 NSCLC cell lines. Gain-of-function and loss-of-function studies showed that overexpression of miR-373-3p promoted H1299 cell migration and invasion, which resulted in upregulation of MMP-9 and MMP-14. By contrast, miR-373-3p knockdown inhibited these processes in A549 cells and downregulated the expression of MMP-9 and MMP-14.

**Conclusion:**

Our results demonstrated that miR-373-3p participated in the invasion and metastasis of lung adenocarcinoma cells, partly by upregulation of MMP-9 and MMP-14.

肺癌位居全球男性和女性癌症病死率的首位，且呈逐年上升的趋势^[1]^。肺癌主要分为小细胞肺癌和非小细胞肺癌，其中非小细胞肺癌是主要的发病类型，约占85%^[2]^。在非小细胞肺癌中，以肺腺癌的发病率最高。肺癌早期往往无明显症状，56%的肺癌患者在确诊时即伴有远处转移，仅15%的患者在病灶局限时被发现^[2]^，因此肺癌细胞侵袭与转移的研究成为临床亟待解决的问题。近年来越来越多的研究发现肺癌侵袭转移的发生与microRNA有密切关系。

MicroRNA是生物体内一类长度约为20个-23个核苷酸的非编码小RNA，是由具有发夹结构的约70个-90个碱基大小的单链RNA前体(pre-miRNA)经过Dicer酶加工后生成的成熟体microRNA-3p和microRNA-5p，通过靶向结合mRNA的3’端非编码区(3’untranslated regions, 3’UTR)，影响目的基因的蛋白表达水平，从而影响细胞的增殖、分化、凋亡与侵袭转移等生物学行为^[3]^。MicroRNA已被证实在多种肿瘤的转移形成中发挥重要作用，包括乳腺癌、肝癌、前列腺癌和结肠癌等^[4]^。

MiR-373是miRNAs-371-372-373家族的成员之一，miR-371/miR-372/miR-373的前体都是相同的，均由染色质19q13.42转录而来^[5]^。MiR-373的前体(pre-miR-373)在细胞质中经过Dicer酶加工后生成成熟体miR-373-3p(即miR-373)和miR-373-5p(即miR-373*)，然而miR-373*在自然界中极少存在。MiR-373在不同的肿瘤中的作用不尽相同，具有组织特异性，扮演着癌基因和抑癌基因的角色，影响着肿瘤细胞的侵袭转移、增殖与周期等生物学行为。研究表明miR-373在乳腺癌^[6]^、人纤维肉瘤^[7]^及胰腺癌^[8]^中对肿瘤细胞的侵袭转移能力有重要调控作用。然而miR-373在肺癌中的作用机制尚未十分明确，为了探讨其与肺癌的关系，本研究通过检测成熟体hsa-miR-373-3p(又称miR-373)在非小细胞癌中的表达情况，同时比较在肺腺癌细胞系中过表达及干扰miR-373-3p后对肺腺癌细胞侵袭转移能力的影响。

## 1 材料与方法

### 1.1 材料

人肺腺癌细胞株A549、H1299、H1975、SPC-A1、PC9和正常支气管上皮细胞HBE均由本实验室提供；非小细胞肺癌组织标本51例和癌旁组织标本39例收集于广东医学院附属医院；RNA提取剂Trizol、反转录及荧光定量PCR试剂盒购自TAKARA；PCR引物合成及细胞培养用的1640培养基购自Invitrogen；胎牛血清(FBS)购自BI；miR-373-3p mimics和miR-373-3p inhibitor购自GenePharma；Lipofectamine^®^ 3000 Transfection Reagent和OPTI-MEN减血清培养基购自Life Technologies；24孔transwell小室(孔径8 μm)购自Corning Costar；侵袭实验所用基质胶(Matrigel)购自BD；基质金属蛋白酶-9(matrix metalloproteinase-9, MMP-9)和MMP14单克隆兔抗人抗体购自CST；RIPA裂解液、BCA蛋白定量及超敏发光试剂盒购自康为世纪公司。

### 1.2 方法

#### 1.2.1 实时荧光定量PCR(qRT-PCR)

检测肺癌组织和肺癌细胞株中的miR-373-3p收集肺癌标本，冰上研磨成粉末状后加入Trizol提取总RNA，应用PrimeScript®miRNA cDNA Synthesis Kit将microRNA反转录成cDNA，通过荧光定量PCR法检测肺癌组织miR-373-3p的相对表达水平。收集处于对数生长期的肺腺癌细胞，PBS洗两遍，加入Trizol提取总RNA，反转录后进行qRT-PCR检测。Has-miR-373-3p和内参U6的上游引物序列分别为：5’-GAAGTGCTTCGATTTTGGGGTGT-3’和5’-CTCGCTTCGGCAGCACA-3’，其共同下游引物Uni-miR qPCR已包含在TAKARA试剂盒内。应用罗氏LightCycler^®^进行实时荧光定量PCR检测。每孔10 μL体系，设置3个平行样，U6作为内参。样本经过3次独立重复实验，所得数据使用2^-ΔCt^或者2^-ΔΔCt^(ΔCt=Ct_目的基因_-CtU6)进行相对定量分析。

#### 1.2.2 细胞转染

将处于对数生长期的A549和H1299细胞接种于6孔板中，待细胞生长汇合度达60%-70%时，利用lipo3000转染试剂将hsa-miR-373-3p mimics及其阴性对照(negative control, NC)瞬时转染至H1299细胞中，将hsa-miR-373-3p inhibitor和NC瞬时转染至A549细胞中。转染时换为无血清无双抗的1640培养基，6 h后换成含10%FBS的培养基，转染48 h后，收集细胞，提取RNA，用qRT-PCR法检测转染前后细胞中miR-373-3p及*MMP-9*、*MMP-14*基因mRNA的改变。转染序列如下：hsa-miR-373-3p mimics：sense 5’-GAAGUGCUUCGAUUUUGGGGUGU-3’，anti-sense 5’-ACCCCAAAAUCGAAGCACUUCUU-3’；mimics NC：sense 5’-UUCUCCGAACGUGUCACGUTT-3’；anti-sense 5’-ACGUGACACGUUCGGAGAATT-3’；hsa-miR-373-3p inhibitor：5’-ACACCCCAAAAUCGAAGCACUUC-3’，inhibitorNC：5’-CAGUACUUUUGUGUAGUACAA-3’。MMP-9上游引物：5’-GCAATGCTGATGGGAAACCC-3’；MMP-9下游引物：5’-AGAAGCCGAAGAGCTTGTCC-3’。MMP-14上游引物：5’-ATCTGCCTCTGCCTCACCTA-3’；MMP-14下游引物：5’-AAGCCCCATCCAAGGCTAAC-3’。

#### 1.2.3 迁移实验

取对数生长期的H1299和A549细胞接种于6孔板，转染48 h后，以每孔200 μL的0.25%胰酶消化细胞，用无血清的1640培养基重悬并调整细胞密度为3×10^5^/mL，取100 μL细胞悬液加入24孔transwell小室的上室，下室预先加入500 μL含10%FBS的1640培养基，放置37 ℃、5%CO_2_培养箱培养24 h后取出小室，用PBS洗两遍，甲醇固定10 min，结晶紫染色10 min。用棉签小心搽拭小室上层细胞，将小室纤维膜取下，用中性树脂将膜封于载玻片中，100倍显微镜下取6个随机视野进行计数。

#### 1.2.4 侵袭实验

取对数生长期的A549和H1299细胞接种于6孔板，转染48 h后，以每孔200 μL的0.25%胰酶消化细胞，用无血清的1640培养基重悬并调整细胞密度为3×10^5^/mL，取100 μL细胞悬液加入24孔transwell小室的上室。上室预先加入50 μL基质胶，室温下风干4 h；下室预先加入500 μL含10%FBS的1640培养基。于37 ℃、5%CO_2_培养箱培养24 h后取出小室，用PBS洗两遍，甲醇固定10 min，结晶紫染色10 min。用棉签小心搽拭小室上层细胞，将小室纤维膜取下，用中性树脂将膜封于载玻片中，100倍显微镜下取6个随机视野进行计数。

#### 1.2.5 Western blot检测转染后细胞内MMP-9、MMP-14蛋白表达水平

收集转染72 h后的的A549和H1299细胞，加入RIPA裂解液和蛋白酶抑制剂提取总蛋白，BCA蛋白定量法检测蛋白浓度，进行SDS-PAGE电泳，将电泳分离的蛋白转至PVDF膜上，5%BSA室温封闭1 h，分别加入1:1, 000兔抗人MMP-9及MMP-14抗体，4 ℃孵育过夜，TBST洗3次，每次10 min，加入辣根过氧化酶偶联的抗兔二抗(1:1, 000稀释)，置于室温摇床1 h，TBST洗膜3次，每次10 min，加入ECL发光液曝光显影，以β-actin为内参，用Quantity One软件分析结果^[9]^。

#### 1.2.6 统计学方法

所有试验均重复3次，采用SPSS 17.0统计软件分析数据。数据均用Mean±SD表示，两组样本间比较用*t*检验，多组样本间比较用单因素方差分析，*P* < 0.05为差异有统计学意义。

## 2 结果

### 2.1 MiR-373-3p在肺癌组织中的表达水平

用qRT-PCR法检测miR-373-3p在51例NSCLC组织和39例癌旁组织中的表达，与癌旁组织相比，miR-373-3p在非小细胞肺癌组织中高表达(*P*=0.011, 9)(图 1)，然而，miR-373-3p在腺癌与鳞癌中的表达无明显差异(*P*=0.904, 9)。有淋巴结转移的肺癌组织中miR-373-3p的表达是无淋巴结转移的3.7倍(*P*=0.008, 7)(图 1，表 1)，这说明miR-373-3p可能与肿瘤细胞的转移能力相关。

**1:**
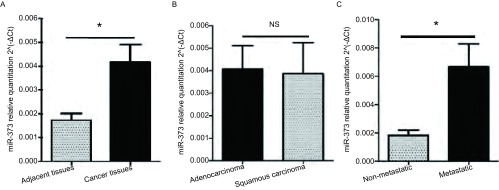
qRT-PCR检测miR-373-3p在肺癌标本中的表达。A：MiR-373-3p在肺癌组织(*n*=51)中较癌旁组织(*n*=39)高表达(*P*=0.011, 9)；B：MiR-373-3p在肺腺癌和鳞癌间的表达差异无统计学意义(*P*=0.904, 9)；C：MiR-373-3p在有淋巴结转移的癌组织中较无淋巴结转移的癌组织高表达(*P*=0.008, 7)。 qRT-PCR of miR-373-3p expression in lung cancer tissue. A: The expression of miR-373-3p was up-regulated in lung cancer; B: MiR-373-3p was not sinificantly different between adenocarcinoma and squamous carcinoma; C: Higher expression was shown in lung cancer with lymph node metastasis. ^*^: *P* < 0.05; NS: *P* > 0.05.

**1:** 非小细胞肺癌患者的临床病理特征与miR-373-3p表达的关系 Clinicopathologic features of non-small cell lung cancer patients and the expression of miR-373-3p

Clinicopathologic features	Case number	∆Ct (Mean+SD) miR-373-3p	*P* value
Age (yr)			0.188
< 60	23	8.31±0.34	
≥60	28	8.98±0.33	
Gender			0.058
Male	31	8.40±1.17	
Female	20	9.39±0.78	
Smoking history			0.174
Yes	26	7.77±1.21	
No	25	8.68±1.42	
Pathological type			0.420
Squamous carcinoma	24	9.11±1.85	
Adenocarcinoma	27	8.54±1.76	
Cell differentiation			0.271
Well	15	8.32±1.30	
Moderately	16	9.16±1.35	
Poorly	20	8.33±1.26	
Tumor stage			0.014
T1-T2	30	9.16±1.36	
T3-T4	21	7.50±1.35	
Lymph node metastasis			0.004*
Present	28	7.88±1.58	
Absent	23	9.45±0.98	

### 2.2 MiR-373-3p在肺腺癌细胞株中的表达

我们进一步检测miR-373-3p在肺腺癌细胞株中的表达情况(图 2)，结果显示与正常支气管上皮细胞(HBE)相比，肺腺癌细胞株(H1299、A549、H1975、SPC-A1、PC9)中miR-373-3p的表达均明显上调(*P*=0.005, 8)。根据肺腺癌细胞中miR-373-3p的表达情况，我们选择在miR-373-3p相对表达水平最低的H1299细胞中过表达miR-373-3p，在miR-373-3p相对表达水平最高的A549细胞中抑制miR-373-3p的表达。

**2:**
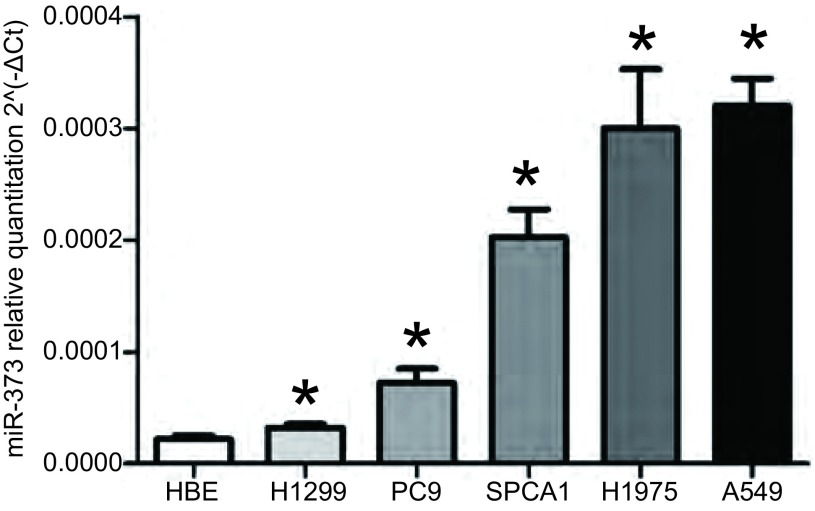
qRT-PCR检测miR-373-3p在肺腺癌细胞株中的表达。MiR-373-3p在5种肺腺癌细胞株中的表达水平较正常支气管上皮细胞明显增高。相比HBE组，^*^P < 0.05。 qRT-PCR of miR-373-3p expression in lung adenocarcinoma cancer cell lines. The expression level of miR-373-3p is sinificantly higher in lung adenocarcinoma cancer cell lines (*n*=5) than in normal bronchial epithelial cell (HBE). ^*^: *P* < 0.05, when compared with HBE.

### 2.3 转染前后细胞中miR-373-3p及MMP-9、MMP-14基因mRNA水平的改变

用瞬时转染法将miR-373-3p mimics转染至miR-373-3p表达量相对最低的H1299细胞中，与NC组相比，mimics组miR-373-3p(*P*=0.006)和MMP-9(*P* < 0.001)、MMP-14(*P* < 0.001)的表达明显增加；将miR-373-3p inhibitor转染至miR-373-3p表达量相对最高的A549细胞中，与NC组相比，inhibitor组miR-373-3p(*P*=0.019, 2)和MMP-9(*P* < 0.001)、MMP-14(*P* < 0.001)的表达明显下调(图 3)。

**3:**
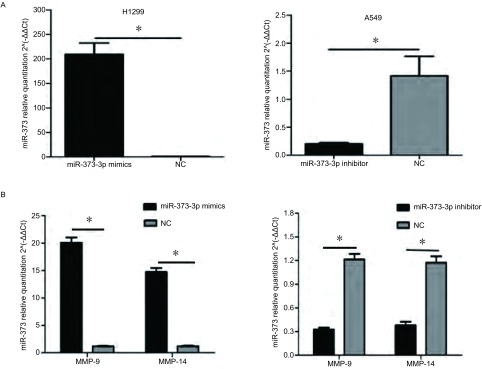
转染后细胞中miR-373-3p、MMP-9和MMP-14基因的mRNA水平。A：瞬转miR-373-3p mimics后H1299细胞中miR-373-3p的表达明显增加，瞬转miR-373-3p inhibitor后A549细胞中miR-373-3p的表达明显减少。^*^：*P* < 0.05；B：过表达miR-373-3p后H1299细胞中MMP-9、MMP-14基因的mRNA明显上调；抑制miR-373-3p表达后A549细胞的MMP-9、MMP-14基因的mRNA明显下调。^*^：*P* < 0.05。 mRNA level of miR-373-3p, MMP-9 and MMP-14 in transfected lung cancer cell lines. A: H1299 cells transfected with miR-373-3p mimics showed an increase in miR-373-3p expression, while A549 cells transfected with miR-373-3p inhibitor resulted in significantly decreased miR-373-3p expression. ^*^: *P* < 0.05 when compared with corresponding negative control. B: mRNA expression of MMP-9 and MMP-14 was up-regulation in H1299 cells with miR-373-3p overexpression, while these genes were down-regulation in A549 with miR-373-3p knock-down. ^*^: *P* < 0.05.

### 2.4 miR-373-3p促进肺腺癌细胞的侵袭与迁移

在Transwell小室迁移实验中，我们发现过表达miR-373-3p后H1299细胞的迁移能力增加1.72倍(*P* < 0.01)，然而抑制miR-373-3p的表达后A549细胞的迁移能力下降54%(*P*=0.012)(图 4A)；与迁移实验结果一致的是，在Transwell小室基质胶侵袭实验中，过表达miR-373-3p后H1299细胞的侵袭能力增加1.62倍(*P* < 0.01)，抑制miR-373-3p的表达后A549细胞的侵袭能力下降45%(*P*=0.03)(图 4B)。

**4:**
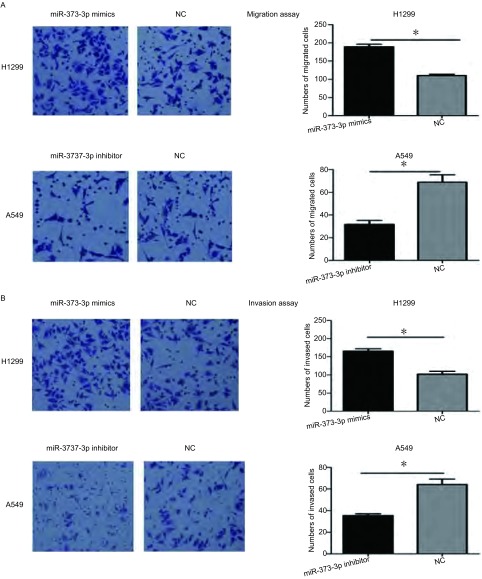
miR-373对肺腺癌细胞迁移与侵袭能力的影响。A：迁移实验。转染miR-373 mimics后促进H1299细胞迁移，转染miR-373 inhibitor后抑制A549细胞迁移；B：侵袭实验。转染miR-373 mimics后H1299细胞的侵袭能力增强，转染miR-373 inhibitor后A549细胞的侵袭能力下降。^*^：*P* < 0.05。 Effects of miR-373 on the migration and invasion of lung adenocarcinoma cancer cells *in vitro*. A: Migration assay. H1299 cells transfected with miR-373 mimics showed an increase in migration capability, while A549 cells transfected with miR-373 inhibitor resulted in significantly decreased migration capability. ^*^: *P* < 0.05 when compared with corresponding negative control. B: Invasion assay. Promotion of invasion ability was shown in H1299 transfected with miR-373 mimics, while A549 cells transfected with miR-373 inhibitor lead to the significantly decrease of invasion ability. ^*^: *P* < 0.05 when compared with corresponding negative control.

### 2.5 改变miR-373-3p的表达对MMP-9、MMP-14蛋白的影响

相比对照组细胞，转染miR-373-3p mimics的细胞中MMP-9、MMP-14蛋白表达上调；与对照组相比，转染miR-373-3p inhibitor的细胞中MMP-9、MMP-14蛋白表达下调(图 5)。

**5:**
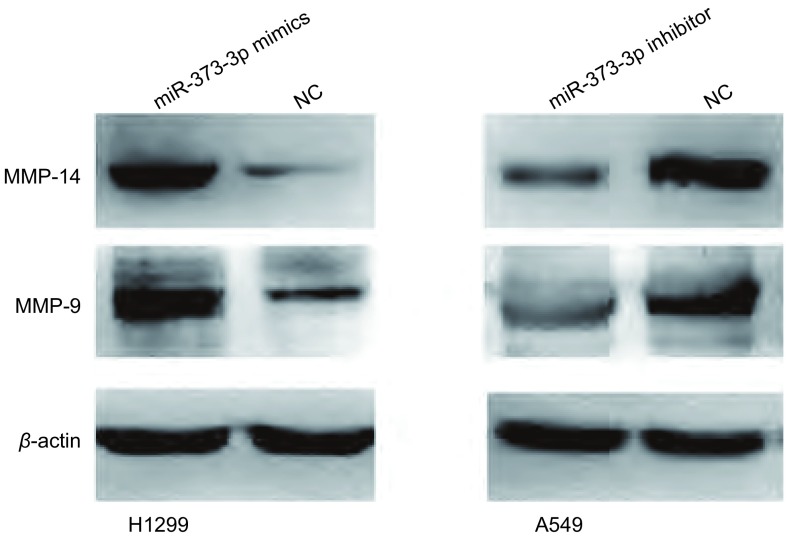
转染后细胞中MMP-9、MMP-14蛋白的表达水平。转染miR-373-3p mimics后H1299细胞中MMP-9、MMP-14蛋白表达上调；转染miR-373-p inhibitor后A549细胞中MMP-9、MMP-14蛋白表达下调。 Expression of MMP-9 and MMP-14 protein in cells after tranfection. Up-regulation of MMP-9 and MMP-14 protein in H1299 cells transfected with miR-373-3p mimics, while down-regulation of MMP-9 and MMP-14 protein in A549 cells transfected with miR-373-3p inhibitor.

## 3 讨论

MiR-373在多种肿瘤中均出现异常表达，扮演着癌基因或者抑癌基因的角色，影响肿瘤细胞的增殖、侵袭和转移过程。有研究^[6]^报道miR-373在乳腺癌中表达上调，通过下调CD44的表达而促进肿瘤细胞的侵袭转移能力。在人纤维肉瘤HT1080细胞中，过表达的miR-373可直接通过靶向结合雷帕霉素靶蛋白(mechanistic target of rapamycin, mTOR)和去乙酰化酶1(sirtuin 1, SIRT1)mRNA的3'UTR而使mTOR和SIRT1的表达下调，进一步导致Ras/Raf/MEK/Erk信号通路和转录因子(nuclear factor kappa B, NF-κB)的激活，从而提高MMP-9的表达水平，增强肿瘤细胞的生长和迁移能力^[7]^。Zhang等^[8]^研究发现miR-373在胃癌中高表达，通过下调靶基因肿瘤坏死因子α诱导蛋白1(tumor necrosis factor, alpha-induced protein 1, TNFAIP1)的表达而促进胃癌细胞的增殖。然而，miR-373与肺癌的研究甚少，两者的关系尚未十分明确。

本研究发现miR-373-3p在非小细胞肺癌组织中较癌旁组织高表达，通过分析研究非小细胞肺癌组织标本的临床病理特征与miR-373-3p表达间的关系发现，有淋巴结转移的非小细胞肺癌组织中miR-373-3p的表达明显高于无淋巴结转移的，另外T3期-T4期的NSCLC组织中miR-373-3p的表达也高于T1期-T2期的，据此我们认为miR-373-3p可能对NSCLC的增殖转移有一定调控作用。而非小细胞肺癌中又以肺腺癌的发病率最高，因此本研究主要探讨miR-373-3p与肺腺癌侵袭转移的关系，进一步研究发现miR-373-3p在5种人肺腺癌细胞株中的表达水平也显著高于人正常支气管上皮细胞，其中以H1299细胞的相对表达水平最低，A549细胞的相对表达水平最高。为了探讨miR-373-3p对肺腺癌细胞的侵袭转移能力的影响，我们通过功能获得以及功能缺失研究发现过表达miR-373-3p可以促进H1299细胞的侵袭转移能力；相反，抑制miR-373-3p的表达可降低A549细胞的侵袭转移能力。以上数据表明miR-373-3p可以促进肺腺癌细胞的侵袭转移能力。与此同时，日本学者Seol等^[9]^在研究miR-373与肺癌的关系时发现过表达pre-miR-373可抑制肺癌细胞的侵袭能力，该研究结果和我们有所不同，存在差异的可能原因是他们的研究中用的是miR-373的前体，而pre-microRNA进入细胞后会在Dicer酶的加工下生成microRNA-5p和microRNA-3p而发挥生物学作用，事实上是否存在microRNA-5p和microRNA-3p的作用差异，这是一个有趣的问题，因为miR-373在不同的肿瘤中也扮演不同的角色，在有的肿瘤中促进转移，有的则是抑制肿瘤转移，例如miR-373促进乳腺癌^[6]^、人纤维肉瘤^[7]^及宫颈癌^[10]^细胞的转移能力，却抑制前列腺癌^[11]^和卵巢癌^[12]^细胞的转移，今后miR-373的亚型在肺癌中的作用仍需进一步研究。

肿瘤侵袭转移的形成涉及多种机制，研究发现基质金属蛋白酶家族(matrix metalloproteinases, MMPs)对于肿瘤转移有重要相关性，他们降解基底膜并在细胞外基质(extracellular matrix, ECM)中暴露出隐藏的多肽抗原，促进侵袭的发生。MMPs可在原发肿瘤处将可溶性肿瘤细胞释放至循环系统中，并在远处器官形成转移微环境以助于随后肿瘤细胞的克隆形成^[13]^。MMPs也可以直接修饰整合素和其他肿瘤细胞粘附分子，激活重要的细胞因子如转化生长因子β(transforming growth factor beta, TGF-β)，诱导上皮-间质转化(epithelial-mesenchymal transition, EMT)的发生，EMT是由增强细胞运动而引起的广泛表型改变，是肿瘤转移的重要过程^[13]^。MMP-9是基质金属蛋白酶家族的成员之一，对肿瘤细胞的生长、增殖和侵袭迁移起着重要作用^[14]^。Peng等^[15]^发现MMP-9高表达与非小细胞肺癌患者的不良预后有密切关系。多项研究^[16-18]^表明MMP-9与增加患者淋巴结转移率、肿瘤远处转移率及缩短无复发生存率有关。MMP-14又称膜型基质金属蛋白酶-1(membrane-type matrix proteinase-1, MT1-MMP)，是第一个被发现的膜型基质金属蛋白酶。MMP-14在正常组织与肿瘤组织的生物学行为中均发挥重要作用，例如侵袭^[19]^、增殖^[20]^与细胞外基质的重塑^[21]^等。研究^[22]^证实相比癌旁组织，MMP-14在非小细胞癌组织中明显高表达。Wang等^[23]^研究发现MMP-14高表达的非小细胞肺癌患者生存时间比MMP-14低表达者更短，是一个不良预后的指标。

为了进一步探讨miR-373-3p促进肺腺癌细胞侵袭转移能力的作用机制，我们对miR-373-3p与基质金属蛋蛋白酶间的关系进行了研究。结果发现过表达miR-373-3p的细胞中MMP-9及MMP-14蛋白的表达水平上调；抑制miR-373-3p的表达后细胞中MMP-9及MMP-14蛋白的表达水平下调。该结果表明，miR-373-3p可能通过上调MMP-9及MMP-14蛋白的表达水平而影响肿瘤细胞的侵袭转移能力。

本研究发现miR-373-3p在肺腺癌中表达上调并扮着癌基因的角色，可能通过上调MMP-9及MMP-14蛋白的表达水平而促进肺腺癌细胞的侵袭转移能力，阐明了肺腺癌细胞发生侵袭转移的机制之一，提示miR-373-3p可能作为抑制肺腺癌细胞侵袭转移能力的靶点，有望为临床上逆转肺腺癌的侵袭转移提供新的思路。
